# A Deadly Liaison between Oxidative Injury and p53 Drives Methyl-Gallate-Induced Autophagy and Apoptosis in HCT116 Colon Cancer Cells

**DOI:** 10.3390/antiox12061292

**Published:** 2023-06-16

**Authors:** Antonietta Notaro, Marianna Lauricella, Diana Di Liberto, Sonia Emanuele, Michela Giuliano, Alessandro Attanzio, Luisa Tesoriere, Daniela Carlisi, Mario Allegra, Anna De Blasio, Giuseppe Calvaruso, Antonella D’Anneo

**Affiliations:** 1 Laboratory of Biochemistry, Department of Biological, Chemical and Pharmaceutical Sciences and Technologies (STEBICEF), University of Palermo, 90127 Palermo, Italy; antonietta.notaro@unipa.it (A.N.); michela.giuliano@unipa.it (M.G.); alessandro.attanzio@unipa.it (A.A.); luisa.tesoriere@unipa.it (L.T.); mario.allegra@unipa.it (M.A.); anna.deblasio@unipa.it (A.D.B.); giuseppe.calvaruso@unipa.it (G.C.); 2 Section of Biochemistry, Department of Biomedicine, Neurosciences and Advanced Diagnostics (BIND), University of Palermo, 90127 Palermo, Italy; marianna.lauricella@unipa.it (M.L.); diana.diliberto@unipa.it (D.D.L.); sonia.emanuele@unipa.it (S.E.); daniela.carlisi@unipa.it (D.C.)

**Keywords:** oxidative stress, phytocompounds, methyl gallate, autophagy, apoptosis, p53

## Abstract

Methyl gallate (MG), which is a gallotannin widely found in plants, is a polyphenol used in traditional Chinese phytotherapy to alleviate several cancer symptoms. Our studies provided evidence that MG is capable of reducing the viability of HCT116 colon cancer cells, while it was found to be ineffective on differentiated Caco-2 cells, which is a model of polarized colon cells. In the first phase of treatment, MG promoted both early ROS generation and endoplasmic reticulum (ER) stress, sustained by elevated PERK, Grp78 and CHOP expression levels, as well as an upregulation in intracellular calcium content. Such events were accompanied by an autophagic process (16–24 h), where prolonging the time (48 h) of MG exposure led to cellular homeostasis collapse and apoptotic cell death with DNA fragmentation and p53 and γH2Ax activation. Our data demonstrated that a crucial role in the MG-induced mechanism is played by p53. Its level, which increased precociously (4 h) in MG-treated cells, was tightly intertwined with oxidative injury. Indeed, the addition of N-acetylcysteine (NAC), which is a ROS scavenger, counteracted the p53 increase, as well as the MG effect on cell viability. Moreover, MG promoted p53 accumulation into the nucleus and its inhibition by pifithrin-α (PFT-α), which is a negative modulator of p53 transcriptional activity, enhanced autophagy, increased the LC3-II level and inhibited apoptotic cell death. These findings provide new clues to the potential action of MG as a possible anti-tumor phytomolecule for colon cancer treatment.

## 1. Introduction

Nowadays, the identification of non-toxic drugs for normal cells capable of selectively targeting tumor systems represents one of the main challenges in the development of innovative and tailored therapies for cancer [1].

Cancer represents a global problem with a continued growing expansion, seriously affecting public health. For many decades, multimodal approaches based on surgery, radiation therapy and chemotherapy have been used. In recent years, a revolution in the development of tumor-targeting drugs has been based on the knowledge of neoplastic entities, which are hallmarks that have substantially improved the types of combinatorial strategies, thus opening the way for precision cancer medicine [2,3].

To achieve this goal, growing interest toward plant-derived chemicals has pushed cancer scientists to search for new unexplored molecules as preventative or anti-tumor compounds. Theoretically, each plant can represent a significant reservoir of bioactive compounds, such as secondary metabolites, phytonutrients, nutraceuticals and supplements [4], that can be obtained from the vegetal world, tested in a laboratory, and applied to ameliorate human health or fight chronic diseases. Plants have developed the ability to synthetize secondary metabolites through highly controlled biochemical pathways that act in response to environmental insults related to abiotic or biotic stresses. In general, these metabolites can be classified as phenolic, terpenoid and alkaloid compounds [5], and due to their multifaceted activities, have stimulated increasing interest from industries for their application as dietary supplements, biocides, pharmaceuticals or potential medicinal drugs.

Due to their intrinsic properties based on the action on multiple targets, limited side effects and excellent efficacies, plant-derived natural products have been applied as healing agents to treat a very wide range of human diseases [6]. A lot of related examples stem from natural drugs used for the treatment of different human malignancies. The main features of these compounds, such as terpenoids (artesunate, atractylodes, andrographolide, etc.), phenols (resveratrol, quercetin, curcumin, capsaicin, etc.) and alkaloids (piperine, berberine, matrine, etc.), rely on their ability to inhibit tumor proliferation and angiogenesis, trigger apoptosis and modulate immune responses [7,8]. A compound that is widely spread in chemotherapeutic regimens is paclitaxel, which is a diterpenoid molecule originally isolated from *Taxus brevifolia* [9,10]. It represents a classical natural phytochemical that has attracted the attention of researchers for its therapeutical ability as an anti-neoplastic agent in the treatment of lung, breast and ovary cancers [10,11,12]. An analog successful chemotherapeutic agent is campthothecin, which is a bioactive compound isolated from *Camptotheca acuminate* [13], that has shown significant efficacy against many different solid tumors when used alone or in combination treatment with cisplatin [14].

In light of these considerations, our research has recently focused on the chemical characterization of plant and fruit extracts or essential oils to discover anticancer phytomolecules to apply alone or as adjuvants to conventional therapies [15,16,17,18,19,20,21,22]. Their identification could also offer the chance to chemically modify plant-derived molecules through the introduction of active pharmacophores, creating novel and powerful lead compounds [23]. In particular, our interest was recently directed toward phytochemicals that are widely found in *Mangifera indica* L., where the peel fraction of the fruit is rich in many bioactive compounds, such as mangiferin; citric acid; quinic acid; digallic acids; gallic acid; and its esters, such as methyl gallate and pentagalloyl glucose [15]. We demonstrated that methyl gallate and pentagalloyl glucose were the most common phytocostituents and were found to be particularly effective in reducing the cell viability of three different colon cancer cell lines [16].

Methyl gallate (MG) is a natural methyl ester of gallic acid and is endowed with many different biological activities ranging from anti-inflammatory to antioxidant and anti-microbial properties [24,25,26,27]. MG was demonstrated to harbor a clear selective anti-neoplastic action in many tumor systems. It plays a crucial inhibitory role in the tumor infiltration of CD4+ CD25+ regulatory T cells and its administration was demonstrated to delay tumor progression and survival in an EL-4 lymphoma model [28]. Other studies provided substantial evidence that this phytomolecule exerts anti-tumor activity on glioma cells, inhibiting proliferation and migratory cell ability via the suppression of the ERK1/2, Akt and paxillin phosphorylation signaling pathways [29]. Huang et al. reported that MG can also exhibit antitumor potential in different HCC cells (Hep3B, Mahlavu and HepJ5) triggering ROS-mediated and caspase-dependent apoptotic cell death [30]. Additionally, MG was also shown to exert a tumor inhibitory effect in in vitro and in vivo mouse models of hepatocellular carcinoma. Furthermore, it did not exert any cytotoxic effect in human normal hepatocytes, while it significantly suppressed the migration, invasion and epithelial–mesenchymal transition in tumor systems of HCC via the AMPK/NF-κB pathway [31].

In light of these observations, and since the anti-neoplastic potential of MG has not been described in colon cancer systems, we explored its possible impact on this tumor. Our investigations provided evidence that this phytochemical starkly reduces colon cancer cell viability, sparing differentiated Caco-2 cells, which provide a model of polarized cells resembling enterocytes [32]. In addition, MG triggered both autophagy and apoptotic cell demise via intertwined crosstalk between oxidative stress and p53 activation as prime sources of the phytochemical action.

## 2. Materials and Methods

### 2.1. Cell Cultures and Chemicals

The Caco-2 and HCT116 colon cancer cells used in this study were obtained from Interlab Cell Line Collection (ICLC, Genoa, Italy) and cultured as monolayers in DMEM supplemented with 10% (*v*/*v*) heat-inactivated FCS and 2 mM glutamine and in the presence of a 1% penicillin/streptomycin solution. To obtain differentiated Caco-2 cells as enterocyte-like cells [33], Caco-2 cells were plated and cultured in a complete medium for 21 days as reported by Natoli et al. [34].

For the reported experiments, cells were seeded in a culture medium on 96-well microplates or 6-well plates as previously reported [16] and allowed to adhere overnight at 37 °C in a humidified atmosphere containing 5% CO_2_ followed by treatment with MG or a vehicle only. MG stock solution was prepared in DMSO and stored at −20 °C according to vendor specifications. In each experiment, MG working solutions were prepared in DMEM, never exceeding 0.01% (*v*/*v*) DMSO. The vehicle condition reported in each experiment as control was represented by untreated cells incubated in the presence of the corresponding DMSO volume. All cell culture media and culture reagents were provided from Euroclone SpA (Pero, Italy). All other reagents and chemicals, except where differently indicated, were purchased from Millipore Sigma (Milan, Italy).

### 2.2. Cell Viability Assay

Cell viability was assessed using an MTT assay as previously reported [35]. Cells were plated in 96-well plates, and after 24 h, they were incubated with compounds for indicated periods. Since the measure of cell viability using an MTT assay is based on the reduction of MTT to formazan and many polyphenols may interfere with formazan production, we considered this aspect in our experimental conditions. In particular, after incubation with the compounds, the plate was centrifuged and the medium was withdrawn and replaced with a fresh one before proceeding with the assay. Afterward, 20 μL of 5 mg/mL MTT was added to each well and the plate was incubated at 37 °C for 2 h. Therefore, the media was removed from each well and replaced with 100 μL lysis buffer (20% SDS and 10% dimethylformamide) before reading at 450 nm. For the determination of IC_50_ values non-linear regression analysis with the equation of a sigmoidal dose response with a variable slope was performed using Graphpad Prism 7.0 software (San Diego, CA, USA).

The observation of cellular morphological changes was detected using a Leica DMR inverted microscope (Leica Microsystems, Wetzlar, Germany), while the pictures were taken using IM50 Leica software (Leica Microsystems, Wetzlar, Germany).

The cytotoxic action of MG was also evaluated using the LDH (lactate dehydrogenase) assay, which is a method based on the measure of the activity of a stable cytoplasmic enzyme commonly released upon cell damage. Cells were seeded in a 6-well plate at a density of 2 × 10^5^ cells, and after incubation with the compound, they were collected and centrifuged at 120× *g* for 10 min. The supernatant medium of each sample was recovered and analyzed using ARCHITECT Lactate Dehydrogenase kit (Abbot Laboratories Diagnostics Division, IL, USA) according to vendor specifications. To detect the total LDH release, treated cells were compared with a positive control represented by cells incubated in the presence of 0.1% Triton 100×.

### 2.3. Colony Formation Assay

This assay measures cell proliferation in a cell-contact-independent way. Cells were plated in pre-tested appropriate densities yielding 500 cells per plate. The plates were cultured for 10 days in the presence or absence of different doses of MG. Then, the colony signals were measured after crystal violet staining as previously reported [15]. The clonogenic survival fraction was defined as the ratio of the signal intensity of the untreated group versus the MG-treated group. All assays were made in triplicate. The number of colonies for each experimental condition was determined using the “Colony Area’’ plugin for the open-source image analysis software ImageJ v 1.8.0 as reported by Guzman et al. [36].

### 2.4. ROS Measurement

To assess the intracellular generation of reactive oxygen species (ROS), cells were plated in 96-well plates and allowed to adhere overnight. Cells were treated with MG and incubation with 5-(and-6)-carboxy-2′,7′-dichlorodihydrofluorescein diacetate (H2DCFDA) fluorochrome (Molecular Probe; Thermo Fisher Scientific, Inc., Life Technologies Italia, Monza, Italy) was performed as previously reported [37]. For this purpose, stock solutions of H2DCFDA were dissolved in DMSO and aliquots were stored at −20 °C until use. H2DCFDA working solution was prepared in a PBS solution containing 5 mM glucose and added to cells to the final concentration of 20 μM. Then, incubation was protracted for 30 min in the dark and ROS-positive cells were visualized using a fluorescein isothiocyanate (FITC) filter (excitation wavelength of 485 nm and emission wavelength of 530 nm) in a Leica inverted fluorescence microscope (Leica Microsystems S.r.l, Wetzlar, Germany) equipped with a DC300F camera. All pictures were captured using Leica Q Fluoro software (Leica Microsystems S.r.l, Wetzlar, Germany).

### 2.5. Measurement of Intracellular Calcium Levels

The intracellular calcium levels were assayed using the Ca^2+^-sensitive fluorescent dye Fluo 3-AM following vendor instructions (Thermo Fisher Scientific, Ferentino, Italy). After incubation with the compounds, cells (2 × 10^5^/well) were collected, washed in calcium-free PBS and incubated in the presence of Fluo 3-AM for 1 h at 37 °C in the dark. Then, calcium generation was analyzed using flow cytometry on a FACSAria Cell Sorter (BD Biosciences Company, 283 Franklin Lakes, NJ, USA). At least 50,000 cells were analyzed for each experimental condition. The data analysis was then performed using FlowJo software workspace v10 (BD Biosciences).

### 2.6. Analysis of Autophagic Vacuoles

The generation of autophagic vacuoles was detected using monodansylcadaverine (MDC) staining according to Munafò et al.’s procedure [38]. Briefly, following to the treatment in the presence of MG, the medium was replaced and 50 mM MDC in PBS was added to cells. Then, cells were washed in PBS and the fluorescence was analyzed with a Leica fluorescence microscope (Leica Microsystems, Wetzlar, Germany) using a 4′,6-diamidino-2-phenylindole dihydrochloride (DAPI) filter (excitation wavelength of 372 nm and emission wavelength of 456 nm). The analysis of autophagic vacuoles was also performed using acridine orange (AO) staining that specifically detects acidic vesicular organelles (AVOs) producing a bright red fluorescence, whereas it generates a bright green fluorescence for cytoplasm and nucleus [39]. For these analyses, cells were incubated for 15 min with 1 μg/mL AO prepared in PBS. Then, cells were analyzed under a Leica fluorescence microscope equipped with an image system (Leica Microsystems, Wetzlar, Germany) using Rhodamine (excitation wavelength of 596 nm and emission wavelength of 620 nm) and FITC (excitation wavelength of 485 nm and emission wavelength of 530 nm) filters. Merged images were obtained by combining pictures of both channels using Leica Q Fluoro software (Leica Microsystems, Wetzlar, Germany).

### 2.7. Immunoblot Analyses

Protein analysis was performed via a Western blotting procedure as previously reported [40]. For these analyses, 30 μg protein/lane were resolved using SDS-PAGE and then electroblotted on a nitrocellulose membrane filter (Bio-Rad Laboratories Srl, Segrate, Italy). All primary antibodies were purchased from Santa Cruz Biotechnology Inc. (Santa Cruz, CA, USA), except for Protein kinase R-like endoplasmic reticulum kinase (PERK), phospho-PERK, eukaryotic initiation Factor 2α (eiF2α) and anti-caspase-3, which were from Cell Signaling Technology (Cell Signaling Technology Inc., Beverly, MA, USA). Anti-rabbit IgG (H + L) HRP conjugate and anti-mouse IgG (H + L) HRP conjugate (dilution 1:10,000) secondary antibodies were from Promega (Milan, Italy). In all experiments performed, γ-tubulin (diluted 1:1000, Sigma-Aldrich, Milan, Italy) was used as the loading control.

For all analyses, protein band detection was performed with an ECL™ Prime Western Blotting System (Cytiva, Merck KGaA, Milan, Italy) using a ChemiDoc XRS System equipped with the Quantity One software 4.6.6 (Bio-Rad Laboratories, Inc., Hercules, CA, USA).

### 2.8. Analysis of Apoptotic Cell Death Using Hoechst and Annexin V/PI Staining

To detect apoptotic cell death, cells were pre-incubated with Hoechst 33342 (Invitrogen; Thermo Fisher Scientific, Inc.) for 30 min before treatment with compounds. Next, blue nuclei showing condensed or fragmented chromatin were analyzed using fluorescence microscopy (Leica Microsystems, Wetzlar, Germany) as reported [15]. The quantification of apoptotic cell death percentage was determined via flow cytometry analysis using an Allophycocyanin (APC) Annexin V conjugate and propidium iodide (Annexin V-APC/PI) staining. For these experiments, HCT116 cells (2 × 10^5^/2 mL medium) were seeded into 6-well plates and then subjected to treatments with MG. At the end of the treatment, the cells were taken via trypsinization, centrifuged at 120× *g* for 10 min, resuspended in PBS and counted. Next, 10^5^ cells were incubated with Annexin V-APC (BD Pharmingen™ APC Annexin V kit, BD Biosciences, Milan, Italy) and PI (Sigma-Aldrich) in the dark according to the manufacturers’ instructions. At the end of the incubation, the samples were analyzed using a FACSAria Cell Sorter flow cytometer (BD Biosciences Company, 283 Franklin Lakes, NJ, USA), acquiring at least 50,000 cells for each sample analyzed. The data obtained were then examined with FlowJo software (BD Biosciences).

### 2.9. Preparation of Cytosolic and Nuclear Extracts

For the isolation of nuclear and cytosolic fractions, 2 × 10^6^ HCT116 cells were plated in 100 mm cell culture dishes and after incubation with MG, lysates were prepared as previously reported [41]. Briefly, cells were washed in PBS and scraped in a lysis solution containing 250 mM sucrose, 20 mM HEPES, 10 mM KCl, 1.5 mM MgCl_2_, 1 mM EDTA, 1 mM EGTA, 1 mM DTT and protease inhibitor cocktail, pH 7.4. Then, the homogenates were prepared by passing cells through a needle of 25 g on ice for 20 min (10 times) and centrifuging samples at 1000× *g* for 10 min at 4 °C. The pellets were recovered and resuspended in lysis solution, and homogenization was repeated by passing cells through a needle of 25 g for an additional 10 times. Therefore, samples were recentrifuged (1000× *g* for 10 min at 4 °C) and pellets representing the nuclear fraction were resuspended in RIPA buffer (1% NP-40, 0.5% sodium deoxycholate, 0.1% SDS, inhibitors of proteases: 25 μg/mL aprotinin, 1 mM PMSF, 25 μg/mL leupeptin and 0.2 mM sodium pyrophosphate) before proceeding with sonication. The supernatants obtained at the first centrifugation were recentrifuged (10,000× *g* for 30 min at 4 °C) and the supernatants obtained were used as the cytosolic fraction. Proteins from both fractions (nuclear and cytosolic) were quantified using a Bradford assay (Bio-Rad Laboratories, Inc.) and were resolved in a polyacrylamide gel to analyze the p53 cellular localization. To determine the purity of each cellular fraction obtained, GADPH and Lamin B were used as cytoplasmic and nuclear markers, respectively.

### 2.10. Statistical Analyses

The statistical analysis of the data was performed by using GraphPad Prism^TM^ 7.0 software (Graph PadPrism^TM^ Software Inc., San Diego, CA, USA) and data were reported as the mean ± S.E. The significant differences between the control (untreated) vs. treated samples were analyzed by applying Student’s *t*-test, while the analysis of multiple groups of samples was conducted using the ANOVA test. The statistical significance threshold was considered to be *p* < 0.05.

## 3. Results

### 3.1. MG Affected Colon Cancer Cell Viability in a Dose-Dependent Manner

**Figure 1:**
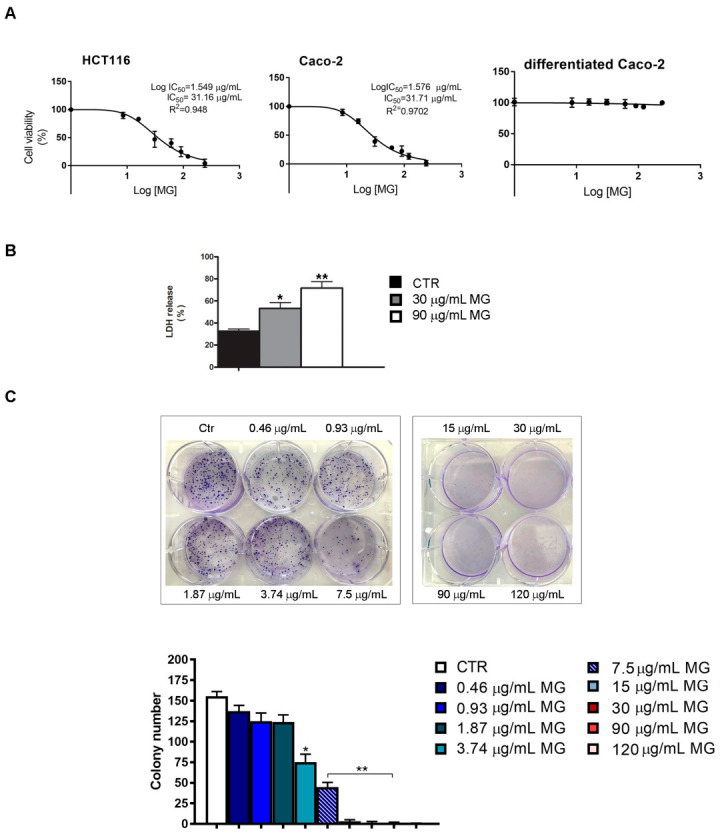
IC_50_ determination and colony formation assay of MG-treated colon cancer cells. (**A**) MTT assay of colon cancer cells (HCT116 and Caco-2) and differentiated Caco-2 cells incubated with incremental doses of MG. Cell viability was determined at 48 h as reported in the Materials and Methods section. IC_50_ values were assessed using GraphPad Prism 7 software. (**B**) LDH cytotoxicity test in MG-treated HCT116 cells. After incubation in the presence or absence of the phytocompound, cells were centrifuged and supernatants were used to assess the LDH content using a commercial kit. Data are reported as a percentage of the total LDH released from cells using as a positive control of cells incubated with 0.1% Triton 100×. (**C**) Clonogenic assay in MG-treated HCT116 cells. The colony formation inhibition was assessed by crystal violet staining after 10 days of exposure to MG. Representative images of colony formation are reported in the upper panel. The quantitative analysis of colonies (lower panel) was performed as reported in the Materials and Methods section. All experiments were performed in triplicate. (*) *p* < 0.05 and (**) *p* < 0.01 compared with the untreated sample.

Studies on the cytotoxic effects of MG demonstrated that this phytochemical elicits remarkable cell viability inhibition in many tumor systems [29,31]. However, since no data are available for colon cancer, we undertook a study aimed at evaluating the MG antitumor potential on the viability of two colon cancer cell lines (HCT116 and Caco-2). Our results showed that the cytotoxic effects were visible after a lag phase of 24 h (not shown) and were clearly evident at 48 h. As can be observed from the response curves (Figure 1A) obtained using incremental doses of MG, the half-maximal inhibitory concentration of MG (IC_50_ value) was about 30 μg/mL in both cell lines after 48 h of incubation. A more consistent effect was observed in the presence of the 90 μg/mL dose, which caused a dramatic decrease in cell viability (about −80%). Differently, in comparison to the corresponding colon cancer Caco-2 cells, no cytotoxic effects were found when MG was administered to differentiated Caco-2 cells, which is a well-established model of polarized intestinal cells reproducing typical morphological and biochemical features of enterocytes [42].

The ability of MG in reducing colon cancer cell viability was also confirmed using both the lactate dehydrogenase (LDH) test and clonogenic assay, which is an in vitro survival test that estimates cell ability to maintain a reproductive potential over a prolonged period. As reported in panel B of Figure 1, MG enhanced the LDH release in HCT116 cells relative to the control. In addition, we also observed that the phytochemical reduced the colony-forming ability of colon cancer cells with doses spacing from 0.46 to 7.5 μg/mL range, while no colonies were found with higher doses. Based on cell viability tests, all further experiments were performed with HCT116 cells using those MG doses that caused cell reductions of about 50% (30 μg/mL) and 80% (90 μg/mL), respectively.

### 3.2. MG Cytotoxicity Was Mediated by Oxidative Injury, ER Stress and Upregulation of Intracellular Calcium

To clarify the underlying mechanism of MG cytotoxicity, we explored whether the observed cytotoxic effect could be ascribed to the induction of oxidative stress. For this purpose, we used NAC, which is a potent radical scavenger.

**Figure 2:**
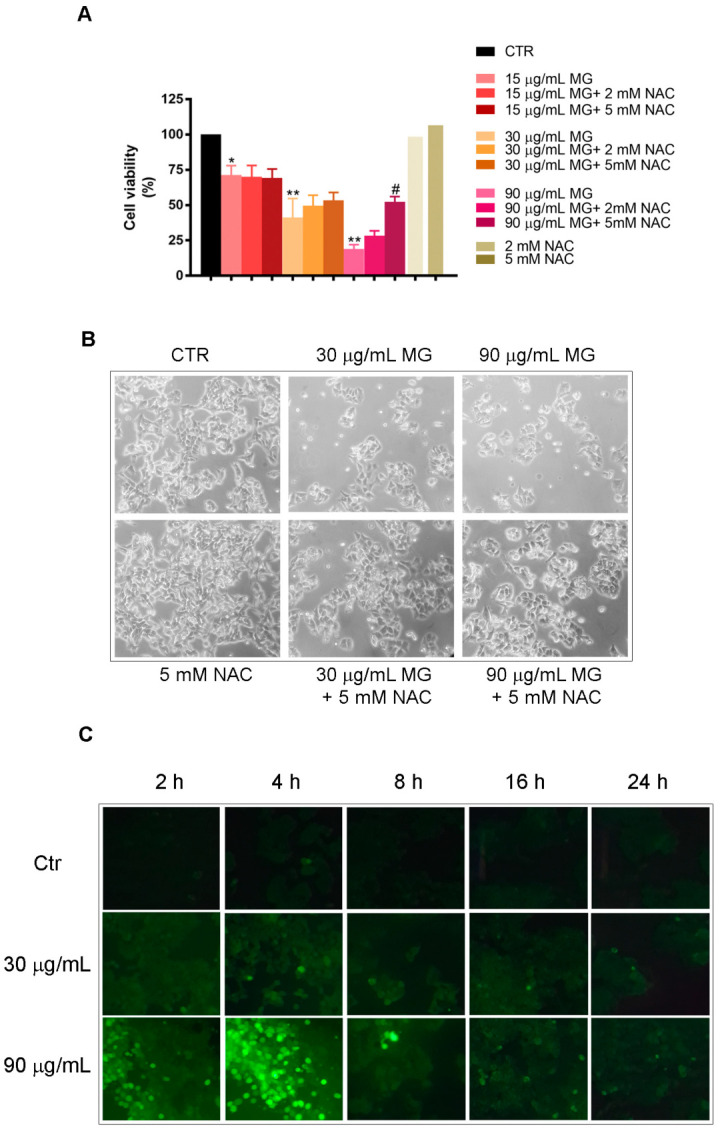
Oxidative stress is required for the cytotoxic efficacy of MG in colon cancer cells. (**A**) Effect of NAC pre-incubation on HCT116 cell viability of MG-treated cells for 48 h. Each value reported in the histogram represents the mean of three independent experiments ± SD. (*) *p* < 0.05 and (**) *p* < 0.01 compared with the untreated sample. (#) *p* < 0.05 compared with the MG-treated sample. (**B**) Phase-contrast micrographs of morphological changes of HCT116 cells treated for 48 h with MG and the protective effect of NAC pre-incubation (original magnification 200×). (**C**) ROS generation induced by MG treatment in HCT116 cells. The ROS level was measured using H2-DCFDA, which is a redox-sensitive fluorescent probe, as reported in the Materials and Methods section. Original pictures were taken using a Leica fluorescence microscope equipped with a CCD camera and FITC filter (original magnification 200×).

As reported in Figure 2A, when pre-incubating the cells in the presence of NAC for 2 h, the toxic effect of 90 μg/mL MG was consistently counteracted. Moreover, all morphological changes induced by MG, consisting of cell shrinkage and a reduction in cell number (Figure 2B), were also counteracted by NAC sustaining the induction of oxidative damage.

Such observations were confirmed by an evaluation of the ROS production assayed using H2DCFDA staining. Following MG exposure at different times, a dose-dependent ROS generation (green fluorescent cells) was observed. The effect, that was already visible after 2 h, reached a peak at 4 h to maintain lower levels for longer times of incubation in the presence of MG (Figure 2C).

**Figure 3:**
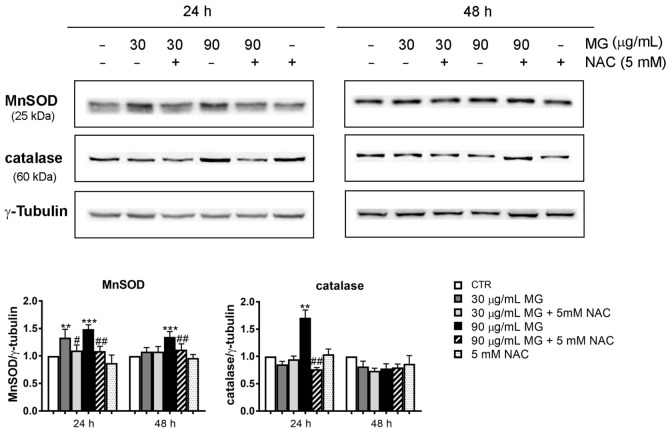
Upregulation of the antioxidant enzymatic systems in HCT116 cells treated with MG. After treatment with the indicated doses of the phytocompound in the presence or absence of NAC, the cell lysates were prepared and the level of stress-associated proteins (MnSOD and catalase) was detected using Western blotting. Representative blots from three independent experiments were considered and a densitometry analysis histogram was normalized to γ-tubulin, which was used as a loading control. (**) *p* < 0.01 and (***) *p* < 0.001 compared with the untreated sample. (#) *p* < 0.05, and (##) *p* < 0.01 compared with the MG-treated condition.

Furthermore, we verified whether these effects were accompanied with the upregulation of stress-associated proteins at 24 h and 48 h. As it can be observed in Figure 3, the manganese superoxide dismutase (MnSOD) and catalase levels, which are two radical scavenger enzymes, increased at 24 h with the two doses of MG. Such an effect was counteracted by the pre-incubation of cells in the presence of NAC (Figure 3). No changes in the levels of these proteins were found at 48 h of treatment.

**Figure 4:**
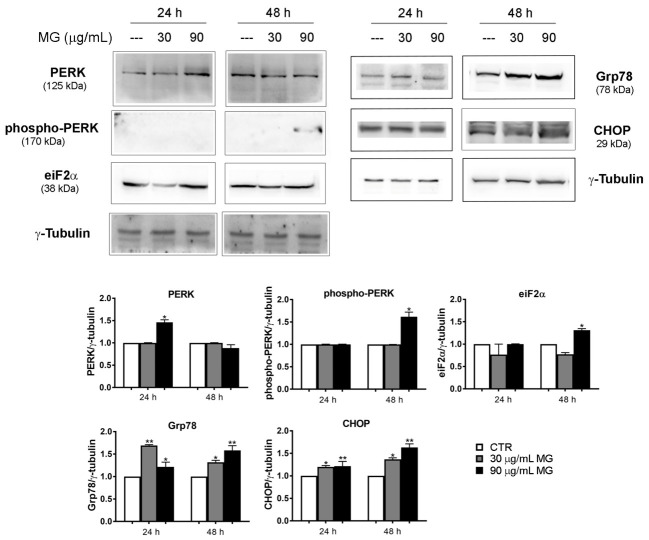
MG exposure activated the ER-stress-associated protein levels. HCT116 cells were treated in the presence of 30 and 90 μg/mL MG for 24 h and 48 h. Western blot analysis was performed to evaluate the protein expression of the ER stress markers PERK, phospho-PERK, eiF2α, Grp78 and CHOP. The amount of analyzed proteins was assessed using γ-tubulin as the loading control protein and for band density normalization. The data are presented as the mean ± SD; (*) *p* < 0.05 and (**) *p* < 0.01 compared with the untreated sample.

In addition, we also investigated whether the oxidative injury triggered by MG exposure could be also associated with ER stress. With this in mind, we conducted Western blotting analyses to explore the status of key factors involved in ER stress. As reported in Figure 4, the higher dose of MG provoked a modest increase in the level of PERK, phospho-PERK and eiF2α. Such an effect was also accompanied at 24 and 48 h with an upregulation of Glucose-Regulated Protein 78 (Grp78), which is an ER chaperone acting as a key regulator of the unfolded protein response (UPR) [43], as well as that of C/EBP Homologous Protein (CHOP).

As it is well known, another event that can contribute to oxidative injury and lead to cell death is a calcium surge released from different cellular compartments, such the ER and mitochondria [44,45,46]. On the other hand, the calcium homeostasis that lies at the heart of many cell signaling processes is under redox control.

**Figure 5:**
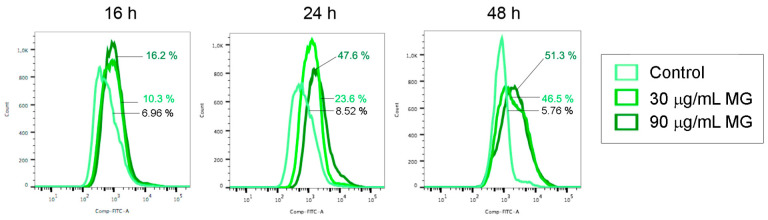
MG exposure increased the intracellular calcium level. Changes in the content of intracellular calcium analyzed at the indicated times via flow cytometry using Fluo 3-AM fluorochrome as reported in the Materials and Methods section.

In accordance with these observations, flow cytometry analyses using a Fluo 3-AM probe provided evidence that the MG provoked a remarkable increase in the intracellular calcium content (Figure 5). Interestingly, this event, which had already occurred at 16–24 h of exposure to MG when cells were found to be still alive, also remained high in treated conditions up to 48 h, a time at which cell death took place, in correlation with the high levels of both Grp78 and CHOP.

### 3.3. Autophagy Was Upregulated in MG-Treated Cells

A growing number of studies indicated that many natural compounds, such as alkaloids, flavonoids, naphthoquinones, sequiterpene lactones and ginsenosides possess an anti-cancer potential acting as autophagy modulators [47]. On the basis of these observations, to further dissect the underlying mechanism of MG, we determined whether this phytochemical might act through the induction of autophagy [48]. Moreover, calcium release from ER storages can also contribute to the generation of autophagosomes [49], which are typical spherical structures endowed with double-layer membranes participating in the autophagic process. Using the monodansylcadaverine (MDC)-based staining, which is a selective fluorescent probe that accumulates in autophagosomes, we observed that MG promoted the generation of autophagic vacuoles, which appeared as dot-like structures.

**Figure 6:**
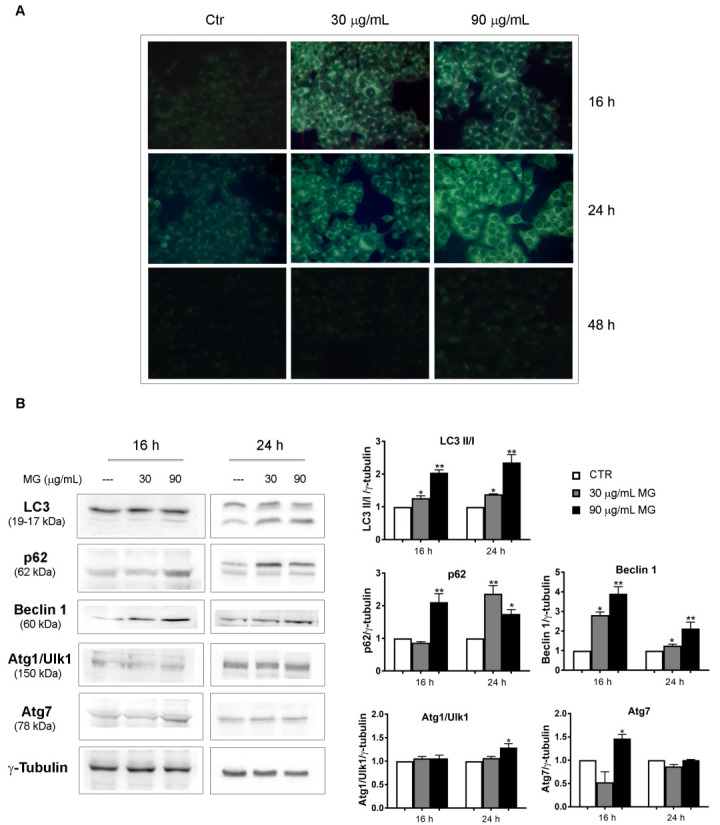
MG exposure provoked an autophagic process in the early phases of treatment. (**A**) Monodansylcadaverine (MDC) staining that enabled the visualization of autophagic vacuoles as dot-like structures was performed in MG-treated cells. HCT116 cells were incubated with MG for the indicated time periods and autophagic vacuoles were highlighted via fluorescence microscopy using a Leica microscope equipped with a DAPI filter. Representative fluorescence microscopy images were taken at a magnification of 400×, as reported in Section 2. (**B**) Immunoblots of autophagic markers performed in MG-treated HCT116 cells. Proteins were detected using different antibodies directed against the LC3-I and LC3-II forms, p62, Beclin 1, Atg1/Ulk1 and Atg7. γ-tubulin was used as the loading control. All graphs show the mean ± SD of three independent experiments. (*) *p* < 0.05 and (**) *p* < 0.01 compared with the untreated cells.

The event, which was already visible at 16 h of incubation, further increased at 24 h, when MDC-positive cells amounted to almost 90% with a 90 μg/mL dose (Figure 6A). Differently, no MDC-stained structures were highlighted at 48 h of treatment.

The induction of autophagy was also sustained by the conversion of microtubule-associated protein 1A/1B-light chain 3 known as LC3-I to LC-3II, which is the phosphatidylethanolamine conjugated form that is recruited to autophagosomal membranes [50,51] and represents a crucial marker of the autophagic flux. An increasing trend of some autophagy-associated factors, such as p62, Beclin 1 and Atg7, was also observed in MG-treated conditions, while no changes were noticed for Atg1/Ulk1 (Figure 6B).

### 3.4. MG Treatment Induced DNA Damage and p53-Mediated Apoptotic cell Death

**Figure 7:**
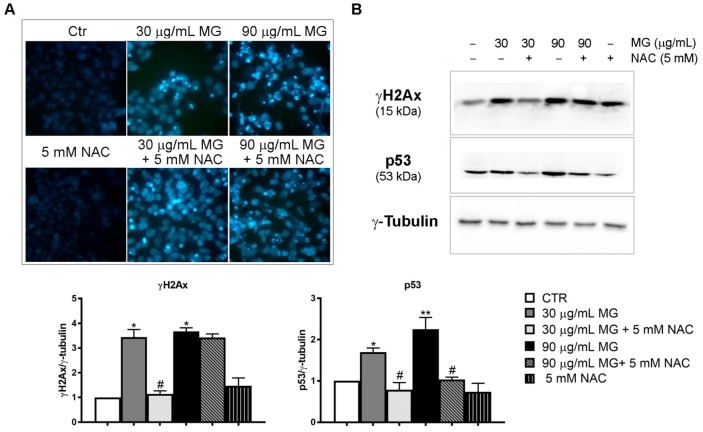
MG-induced DNA damage. (**A**) Morphological analysis of HCT116 cells after vital staining with Hoechst 33342. Following Hoechst staining, cells were treated for 48 h with different doses of MG in the presence or absence of NAC. Chromatin fragmentation and condensation were observed under a fluorescence microscope. The images (original magnification at 200×) were acquired with a DAPI filter using an inverted fluorescence microscope and processed with Leica Q Fluoro Software. (**B**) Analysis of DNA damage markers: γH2AX and p53. After treatment with MG in the presence or absence of NAC for 48 h, cells were lysed and proteins were analyzed using Western blotting. The γ-tubulin blot was reported as a loading control. The blots and histograms of densitometric analyses reported are representative of three independent experiments. (*) *p* < 0.05 and (**) *p* < 0.01 compared with untreated cells. (#) *p* < 0.05 compared with the MG-treated condition.

To evaluate whether the cytotoxic effect observed in the presence of MG could be ascribed to the induction of apoptotic cell death, we tested possible chromatin condensation and fragmentation using vital Hoechst staining. While MG administration triggered autophagy in the first phase of treatment, data reported in Figure 7A showed that when prolonging the exposure up to 48 h, remarkable nuclear modifications associated with cell death occurred in the presence of the phytocompound.

When exploring possible changes in DNA damage markers, we provided evidence that the phytochemical produced a consistent increase in γH2AX, as well as p53 (Figure 7B). Meanwhile, the pre-incubation with NAC counteracted MG effects on both chromatin condensation and γH2AX and p53 activation.

**Figure 8:**
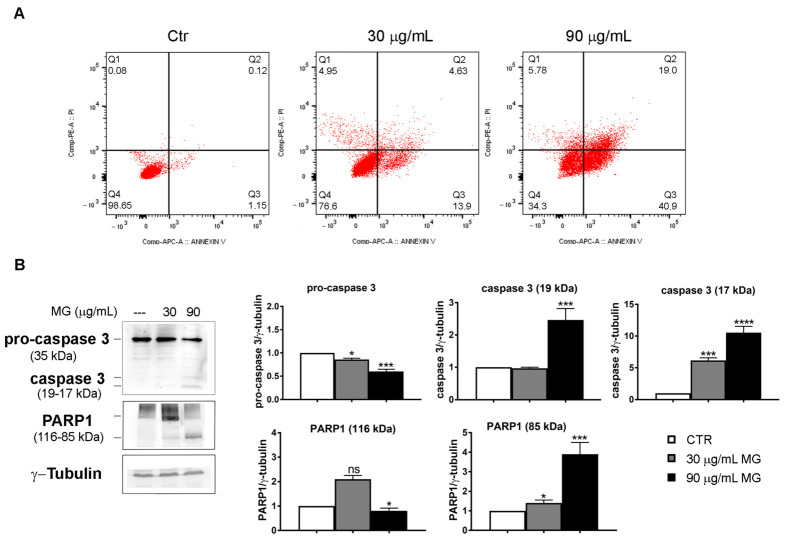
MG-induced apoptosis in colon cancer cells. (**A**) Annexin V/PI staining to evaluate apoptosis. HCT116 cells were treated with MG and compared with untreated cells. The rate of apoptosis was assessed via flow cytometry using the Annexin V/PI double staining assay. The data represent one of three independent experiments. (**B**) MG treatment evoked an increase in apoptotic markers. Caspase-3 activation and PARP1 fragmentation were analyzed using Western blotting. The relative quantification was assessed after densitometric analysis of bands and normalization to γ-tubulin used as a loading control. Histograms of densitometric analyses report the average values of three independent experiments. (*) *p* < 0.05, (***) *p* < 0.001 and (****) *p*< 0.0001 compared with the untreated sample. ns, not significant.

To elucidate the type of cell death induced by MG, we performed flow cytometry analyses using Annexin V/PI double staining (Figure 8A). At 48 h, about 19% of early (Q3 quadrant) and late (Q2 quadrant) apoptotic cells were found using 30 μg/mL of the compound. Such a value amounted to 60% apoptotic cells when the higher dose of MG was employed. We then checked the status of specific apoptotic markers, such as caspase-3 and its target PARP1. Specifically, we found that MG promoted a decrease in the pro-enzymatic form of caspase-3 and the appearance of the fragmented and activated forms at 19 and 17 kDa, respectively. Such an effect was accompanied with the fragmentation of PARP1, which is a caspase-3 target (Figure 8B).

### 3.5. MG Treatment Induced Early Upregulation of p53 Related to the Molecular Switch between Autophagy and Apoptotic Cell Death

**Figure 9:**
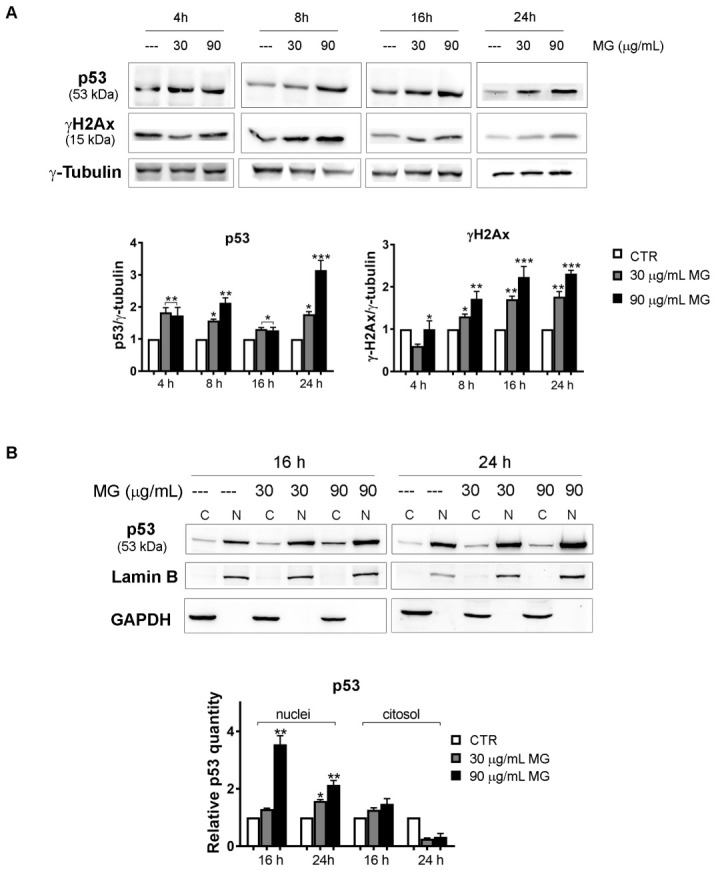
Time course analysis of the MG action on DNA damage markers and p53 nuclear accumulation. (**A**) MG treatment provoked an early upregulation of DNA damage markers p53 and γH2Ax in HCT116 cells. After treatment with MG for various periods, p53 and γH2Ax were detected using Western blotting analyses. Data were normalized to γ-tubulin, which was used as the loading control. The blots and histograms of densitometric analyses reported are representative of three independent experiments. (*) *p* < 0.05, (**) *p* < 0.01 and (***) *p* < 0.001 compared with the untreated sample. (**B**) Subcellular fractionation for cytosolic and nuclear protein extract displayed nuclear enrichment in the p53 content after the MG incubation. The relative quantification was assessed after densitometric analysis of the bands and normalization to the correspondent loading control. Lamin B or GADPH was used to assess the possible changes in the loaded protein amount for the cytosolic and nuclear fractions, respectively. (*) *p* < 0.05 and (**) *p* < 0.01 compared with the untreated sample.

In light of these results, we wondered about the possible role of p53 and γH2AX in the mechanism analyzed. Time course studies (4–24 h) provided evidence that both doses of MG promoted an early upregulation of these factors, which were already visible after 4 h of incubation with the compound (Figure 9A). As it is known, p53 protein is activated in key responses to genotoxic stresses and DNA damage. In these scenarios, p53 translocates to the nucleus, boosting a tumor suppressor program via the cell cycle arrest or apoptosis via the direct transcriptional activation of specific pro-apoptotic targets, such as Apaf-1, Puma, Bax and Noxa [52,53]. By performing subcellular fractionation experiments, we found p53 accumulation in the nuclear fraction already at 16 h of incubation with MG (Figure 9B).

To further dissect the underlying role of p53 since its increase was already observed in the early phases of treatment (4–16 h) when oxidative injury and the autophagy process occurred, we explored a possible interplay between p53 and MG-induced autophagy. Compelling evidence showed that p53 can positively or negatively impact autophagy in a context-dependent manner or via its subcellular localization [54,55,56,57].

**Figure 10:**
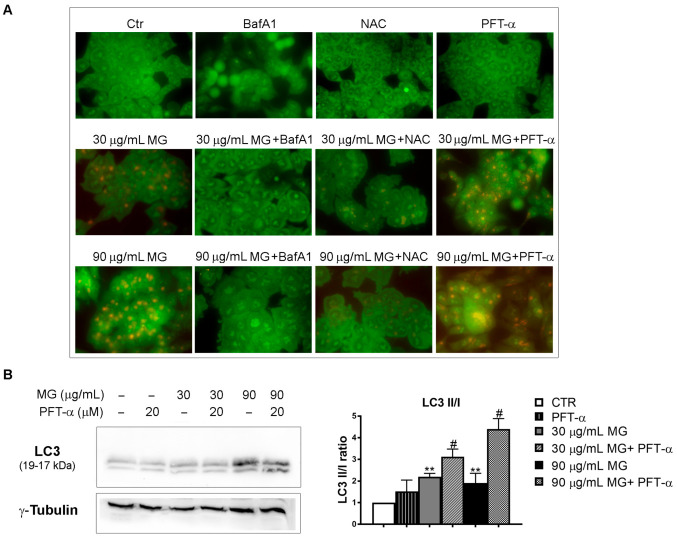
Effects of p53 inhibition on MG-induced autophagy. (**A**) The inhibition of p53 transcriptional activity by PFT-α enhanced the MG-induced autophagy in HCT116 cells. Cells were pre-incubated with NAC (5 mM), BafA1 (100 nM) or PFT-α (20 μM) in the presence or absence of MG for 24 h; then, the production of AVOs showing bright red fluorescence was evaluated via AO staining using Leica Q Fluoro software. (**B**) PFT-α/MG co-treatment enhanced the levels of the autophagic protein LC3. For the analysis of the LC3 forms, cells were treated with MG in the presence or absence of PFT-α (20 μM), followed by a Western blot analysis. The relative quantification was assessed after a densitometric analysis of bands and normalization to γ-tubulin. Data reported in the histograms were the average of three independent experiments. (**) *p* < 0.01 compared with the untreated sample. (#) *p* < 0.05 compared with the MG-treated sample.

By using acridine orange (AO) staining, which corroborated the accumulation of acidic vesicular organelles (AVO, orange fluorescence) in the cytoplasm of MG-treated conditions, we observed that the addition of antioxidant NAC counteracted the autophagy, while Bafylomicin A1, which is an inhibitor of autophagosome-lysosome fusion, markedly inhibited the autophagic process (Figure 10A). On the other hand, pre-incubating the cells with pifithrin-α (PFT-α), which is a specific inhibitor of p53 transcriptional activity, the AVO accumulation induced by MG further increased (Figure 10A). Such an effect was also accompanied by an upregulation of the LC3-II form (Figure 10B), thus suggesting that p53 can negatively affect autophagy in response to MG.

**Figure 11:**
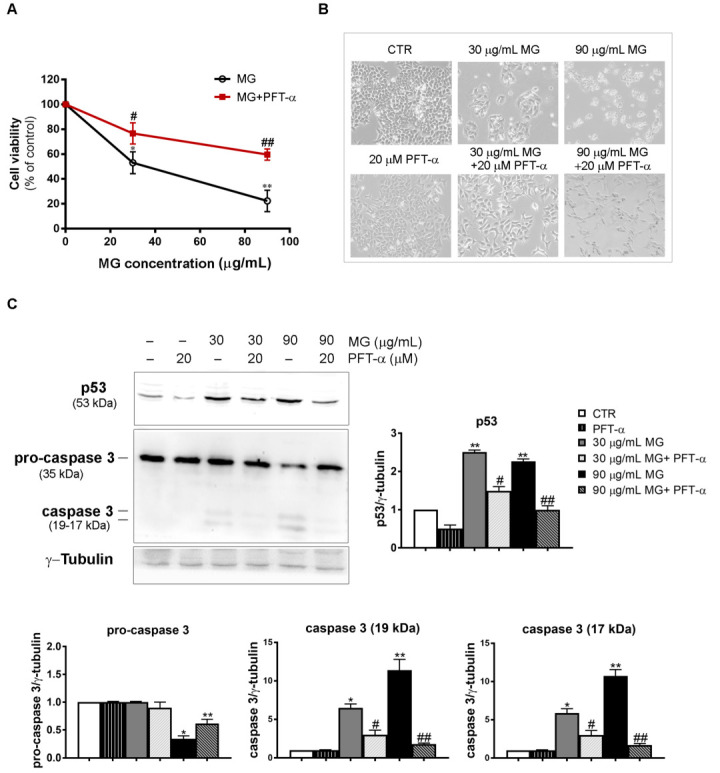
PFT-α, which is a p53 inhibitor, negatively affected the MG-induced apoptosis in HCT116 cells. (**A**) PFT-α inhibited the MG-induced cytotoxic effect. The pre-incubation of cells with PFT-α was performed as reported in the Results section; then, different MG concentrations were added and incubation was protracted for 48 h. Cell viability was analyzed using an MTT assay. Values reported in the line chart represent the mean of three independent experiments ± SD; (*) *p* < 0.05 and (**) *p* < 0.01 compared with the untreated conditions. (#) *p* < 0.05 and (##) *p* < 0.01 compared with the MG-treated sample. (**B**) Micrographs showing the PFT-α effect on morphological changes induced by the MG treatment. Pictures were taken using a Leica inverted microscope as reported in the Materials and Methods section. (**C**) Effect of PFT-α on the p53 and caspase-3 levels. Protein lysates were prepared as reported in the Materials and Methods section and resolved using SDS-PAGE. Blots were detected using specific antibodies directed against the proteins of interest and their level was normalized to γ-tubulin, which was used as the loading control. The blots and histograms of densitometric analyses reported are representative of three independent experiments. (*) *p* < 0.05 and (**) *p* < 0.01 compared with the untreated sample; (#) *p* < 0.05 and (##) *p* < 0.01 compared with the MG-treated sample.

Accordingly, when we pre-incubated HCT116 cells for 2 h in the presence of PFT-α, followed by a co-treatment with MG for 48 h, PTF-α played a protective role against the cytotoxicity showed by the phytocompound alone. As shown in Figure 11A, the residual viability that amounted to only 22% with 90 μg/mL MG rose to about 60% when PFT-α was added.

Such prevention of MG cytotoxic effects was also found when PFT-α was co-administered and morphological changes were evaluated using light microscopy (Figure 11B).

To further ascertain whether PFT-α can inhibit MG-induced apoptosis, we explored its effect on both p53 and caspase-3. The results showed that compared with the control group, PFT-α counteracted p53 upregulation and, in the same experimental conditions, also suppressed caspase-3 activation, inhibiting the production of its active fragments (19–17 kDa) induced by the MG exposure (Figure 11C).

## 4. Discussion

Moderate levels of ROS are crucial regulators of signaling pathways relevant for life in normal cells, while their increased generation can seriously impair cellular redox balance, contributing to many pathologic conditions, such as cancer. Many different studies highlighted that tumor cells, compared with their normal counterpart, exhibit an increased oxidative burst that favors cancer transformation, metabolic remodeling and increased generation of ROS [58]. Indeed, an escalated production of ROS positively impacts cancer through pro-tumorigenic signaling that enhances cell survival, DNA damage, genetic instability, hypoxia adaptation and resistance to the most common chemotherapeutics [59]. In this intricate scenario, malignant cells thrive in and counterbalance the ROS overload by boosting a plethora of enzyme-based scavenger systems to detoxify themselves with an oxidative burst and maintain their pro-tumorigenic profile.

However, despite these aspects, the role of ROS in cancer and anti-cancer strategies is still widely debated. A growing body of evidence supports the view that ROS conceal an oncojanus nature, either activating pro-tumorigenic or anti-tumorigenic signaling. Such pathways can be differently orchestrated in cancer treatment to preferentially kill tumor cells [58,60]. The fine-tuning of some anti-neoplastic therapies that promote an escalated ROS generation that overwhelms the scavenging tumor ability seems to be the other side of the coin that could be exploited as an Achilles’ heel and drive tumors to different death pathways, such as apoptosis, autophagy or necroptosis [61].

Based on these rationales, some chemotherapeutics as natural or synthetic compounds (i.e., platinum-based compounds, anthracyclines, taxanes and sesquiterpenoids) have been extensively used with this purpose to treat tumors [62,63].

In particular, the investigations developed in this study focused on methyl gallate (MG), which is a well-known phytochemical that harbors strong anti-neoplastic properties in many different tumor systems, but no data are available on colorectal cancer. Here, we provide evidence for the first time that MG inhibits the growth of colon cancer cells, sparing differentiated Caco-2 cells, which is a model of polarized enterocytes. In particular, the cytotoxic action of MG could be ascribed to a deadly liaison occurring between ROS and p53 that, unavoidably, dictates the cell fate toward apoptosis. In the first phase of treatment, MG stimulated a stress-associated program characterized by the precocious increase in ROS content, along with an upsurge in both ER stress markers (PERK, phospho-PERK, Grp78 and CHOP) and the intracellular calcium level. As a consequence of this oxidative burst, probably to serve a stress defense response, MG-treated cells also upregulated the level of the ROS scavenger enzymes MnSOD and catalase.

In this complex scenario, we do not know the origin of intracellular calcium increase at this time. As it is well known, changes in intracellular calcium content that increase beyond the normal threshold can be ascribed to its release by the ER or dysfunctional mitochondria. Since some of our preliminary studies also suggested an involvement of mitochondria in MG-induced mechanism, we cannot exclude this possibility and aim to better clarify such an aspect in our future directions.

Overall, the early ROS generation drove cells in the first phases of treatment (16–24 h) along an autophagy process, as testified by the appearance of autophagic vacuoles to MDC and AO staining and significant changes in autophagic markers, such as LC3, p62, Beclin 1 and Atg7. However, when analyzing the timeline of MG exposure up to 48 h, such an autophagic flux was interrupted, leading cells to an apoptotic demise characterized by DNA fragmentation and caspase activation. These findings were in accordance with Huang‘s data [30], demonstrating that the extensive oxidative injury induced by MG treatment is a causative event in the apoptosis triggered by MG in HCC cells. Indeed, in our experimental condition, the addition of NAC, which is an antioxidant sulfidryl compound, prevented the toxic effect of MG in colon cancer cells, inhibited the autophagic process and counteracted the DNA fragmentation occurring during the apoptotic cell death. On the other hand, the observation that oxidative stress could play a role in the analyzed events was shown by data that reported a complex interconnection between ROS production, ER stress and autophagy [64,65].

Interestingly, our results also indicated that a pivotal role in the mode of action of MG was played by the tumor suppressor protein p53. p53 represents a key player that is capable of monitoring a plethora of cellular pathways related to the control of cell cycle progression, genome stability and apoptosis [66]. For all these multifunction properties, it has been named the “guardian of the genome”. Accumulating evidence demonstrated that under normal conditions, p53 is maintained at very low levels by its negative regulator MDM2 that targets p53 degradation to 26S proteasome [67]. Differently, when cells are under stress conditions (nutrient deprivation, hypoxia, DNA damage), the p53 level increases and the protein translocates to the nucleus, where, as a transcription factor, it regulates the expression of a subset of genes functioning in cell cycle progression, cell metabolism, autophagy, tumor microenvironment and apoptosis. ROS and p53 were shown to establish a versatile partnership [68]. Indeed, ROS generated as by-products of cellular metabolism can act either upstream of p53, promoting its expression, or downstream, triggering apoptotic cell death pathways. In our experimental conditions, MG stimulated p53 upregulation, and such an effect seemed to be strictly intertwined with ROS generation. Its increased level, which was already visible in the first hours of exposure to MG (4–8 h), was maintained at a high level up to 48 h, when DNA damage occurred and cells collapsed via apoptosis. On the other hand, the addition of NAC counteracted the increase in both p53 and γH2Ax DNA damage markers, demonstrating that their upregulation can be ascribed to the impairment of redox balance induced by the phytocompound. Clearly, we believe that p53 was crucial in the context of the mechanism studied when monitoring both autophagy and apoptotic cell demise. Such an observation was supported by the experiments performed using PFT-α, which is a specific inhibitor of p53 transcriptional activity. Indeed, when PFT-α was co-administered with MG, we observed an enhancement of autophagy flux, as well as in LC3-II form, showing that p53 could inhibit autophagy in our condition. The relationship between p53 and autophagy has been widely discussed and still appears to be controversial since p53 can act as a rheostat system that adjusts the authophagy rate (through a positive or negative regulation) in a context-dependent fashion [56].

On the other hand, p53 also represents an active player in MG-induced apoptosis in colon cancer cells. Indeed, when exposing cells to a PFT-α/MG combo treatment, we observed that the p53 level dropped and the cytotoxic action of MG, as well as caspase-3 activation, were prevented by PFT-α.

**Figure 12:**
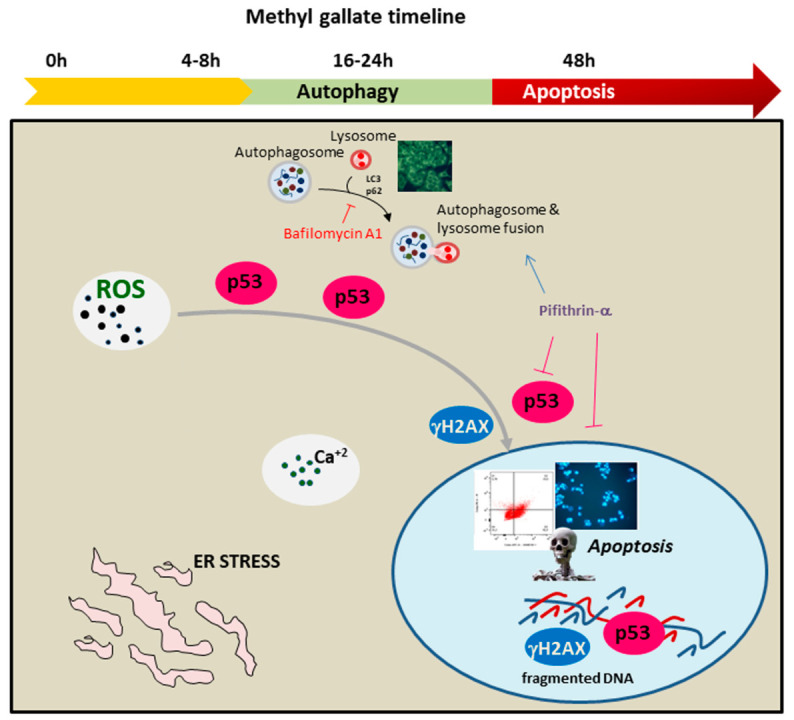
Timeline of the antitumor signaling pathway activated by MG in colon cancer cells. MG exposure triggered autophagy and apoptotic cell demise. The represented processes are orchestrated by an intertwined liaison between oxidative injury and p53. The early generation of ROS, accompanied with intracellular calcium increase and ER stress, stimulated p53 to switch the autophagy toward apoptotic cell death.

A schematic representation describing the intricate mechanism of MG anticancer activity on colon cancer cells is reported in Figure 12.

## 5. Conclusions

As a whole, the findings reported in this paper fit well with the current scientific literature on the anti-tumor potential of methyl gallate. In particular, our results shed light on the potential action of MG in preferentially targeting colon cancer cells with respect to enterocyte-like cell models. The biochemical characterization of MG signaling in these cells reveals a new clue to its underlying mechanism, thus highlighting an intertwined relationship between oxidative stress and p53 as a causative event in apoptotic demise. The data obtained here represent a reason to use MG in future investigations and explore whether this phytochemical can be used alone or in combinatorial studies as a possible adjuvant in traditional chemotherapy.

## Data Availability

Data reported in this paper are available on request from the corresponding author.
